# Magnetically Separable and Recyclable Fe_3_O_4_-Supported Ag Nanocatalysts for Reduction of Nitro Compounds and Selective Hydration of Nitriles to Amides in Water

**DOI:** 10.3390/molecules19010699

**Published:** 2014-01-07

**Authors:** Hyunje Woo, Kyoungho Lee, Sungkyun Park, Kang Hyun Park

**Affiliations:** 1 Department of Chemistry and Chemistry Institute for Functional Materials, Pusan National University, Busan 609-735, Korea; E-Mails: hjwoo@chemistry.or.kr (H.W.); kyoungho@chemistry.or.kr (K.L.); 2 Department of Physics, Pusan National University, Busan 609-735, Korea; E-Mail: psk@pusan.ac.kr

**Keywords:** silver, Fe_3_O_4_ support, reduction, hydration, magnetic separation

## Abstract

As hybrid nanostructures have become more important in many fields of chemistry, Ag nanoparticles (NPs) are being increasingly immobilized onto Fe_3_O_4_ microspheres *in situ*. Structural characterization reveals that the Ag NPs are uniformly immobilized in the Fe_3_O_4_ microsphere-based supports. Moreover, Ag NPs are more stable in the hybrid structure than in the naked state and show high catalytic activity for the reduction of nitro compounds and hydration of nitriles to amides in water. The Fe_3_O_4_ microspheres were recycled several times using an external magnet.

## 1. Introduction

In recent years, numerous attempts have been made toward designing and synthesizing hybrid nanostructures, which combine or even improve the physical and chemical properties of the constituent parts [1]. Many studies have discussed the syntheses of these multicomponent nanostructures with increased functionality [2,3]. The presence of multicomponent functions combined with the enhanced chemical and physical properties make hybrid nanostructures suitable for research fields pertaining to the study of magnetic, plasmonic, and semiconducting properties [4]. Metal NPs immobilized onto metal oxide supports can be used as catalysts in organic reactions, wherein they show higher catalytic activity than the naked metal NPs because of electron transfer across the interface [5,6,7,8].

Among the existing metal oxide supports, Fe_3_O_4_ has gained significance owing to its nanostructure and cutting-edge technological applications, including its usage as a magnetic storage medium [9], in biosensors [10], as well as medical applications such as targeted drug delivery [11,12]. Furthermore, Fe_3_O_4_ microspheres have been used as supports for the immobilization of metal NPs in nanocatalysis because of the ease of their recyclability using external magnets after the reactions [13].

Recently, many researchers have described the preparation of Ag NPs and studied their various applications in highly active surface-enhanced Raman scattering substrates [14], antibacterial coatings [15], electrochemical and biosensors [16], and as efficient catalysts for organic reactions [17]. A significant number of studies on the catalytic reactions of Ag NPs have been conducted, such as alcohol dehydrogenation, oxidation of phenylsilanes, reduction of aromatic compounds, and Diels-Alder cycloadditions [17,18,19,20]. However, during a reaction, the highly active surface atoms destabilize the NPs. To overcome this problem, metal NPs have been immobilized onto supports to increase the stability of the catalysts [21,22].

**Scheme 1:**
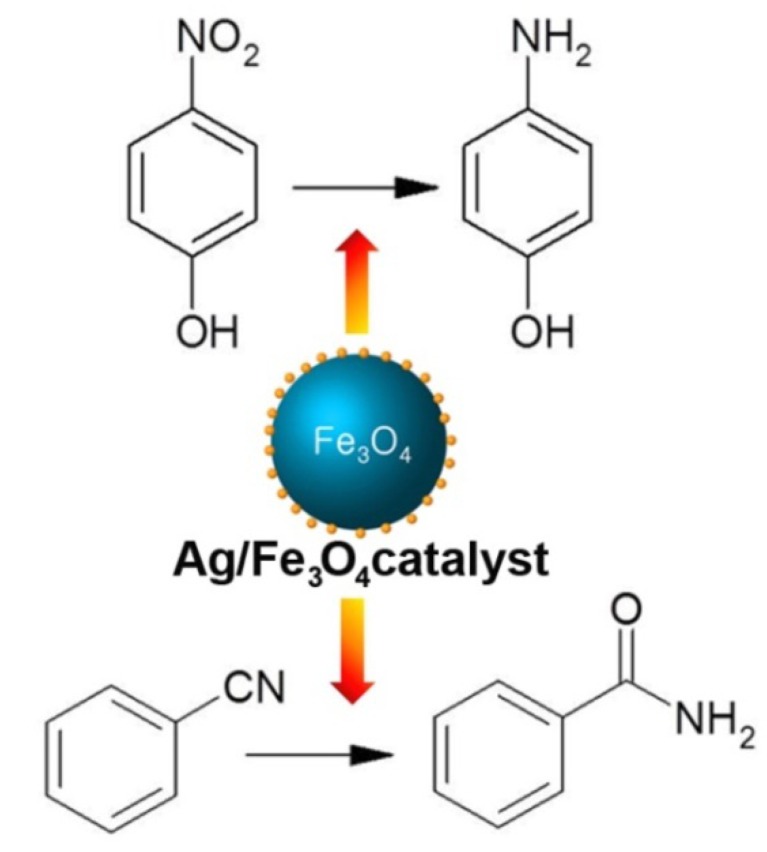
Reduction of a nitro compound and hydration of a nitrile to an amide catalyzed by Ag/Fe_3_O_4_ catalyst.

4-Nitrophenol (Scheme 1) is considered to be one of the most refractory pollutants in wastewaters generated by industrial sources such as companies that manufacture explosives and dyes [23,24]. Due to the importance of 4-aminophenol, there have been many reports about applications of Ag nanoparticles on various supports as catalysts for the reduction of 4-nitrophenol [25,26,27]. Among them, the use of water as a solvent under mild conditions has attracted much attention in the environmental field. Also, the hydration of nitriles to yield the corresponding amides is of tremendous significance to researchers in academia and industry alike because the resulting amides have numerous applications in synthetic organic and pharmaceutical chemistry. Among various heterogeneous nanocatalysts, supported Ag NPs have been used for this reaction as catalyst [28,29]. In our earlier work, Ag NPs were also used as catalysts for the hydration of nitriles to amides [30].

In this paper, Ag NP, immobilized onto Fe_3_O_4_ microspheres were synthesized and tested as a catalyst in reduction of nitro compounds and nitrile hydration reactions in water (Scheme 1). The monodisperse Ag NPs were easily immobilized without any pretreatment such as attaching functional groups onto the Fe_3_O_4_ microspheres. This catalyst showed increased Ag NP stability and could be easily recycled using an external magnet after the completion of the reaction.

## 2. Results and Discussion

### 2.1. Catalyst Preparation and Characterization

**Figure 1:**
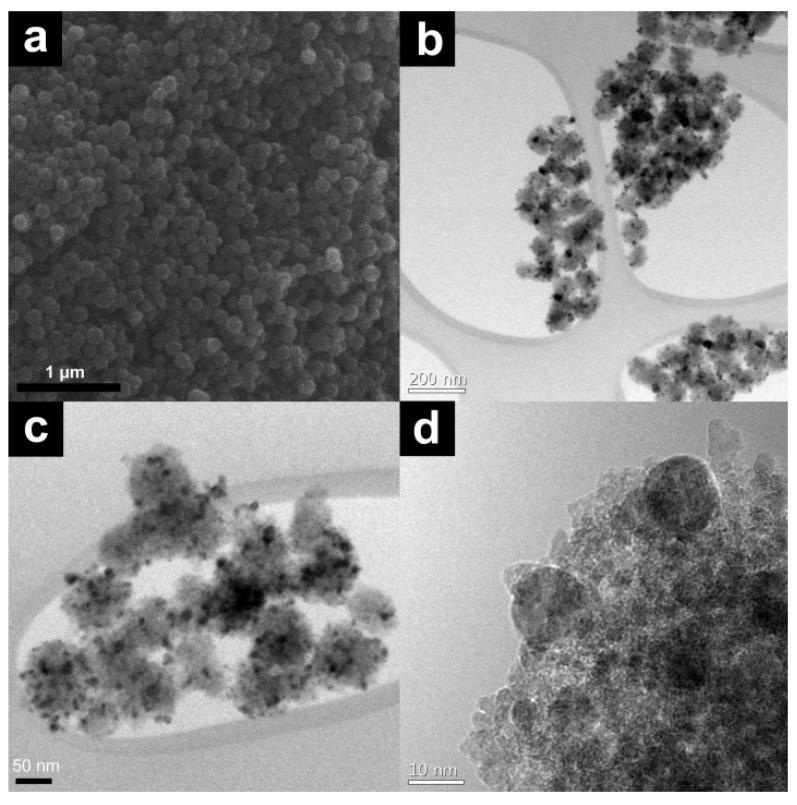
(**a**) SEM image of Fe_3_O_4_ microspheres; (**b**,**c**) TEM images of Ag/Fe_3_O_4_; and (**d**) Immobilized Ag NPs onto Fe_3_O_4_ microspheres.

**Figure 2:**
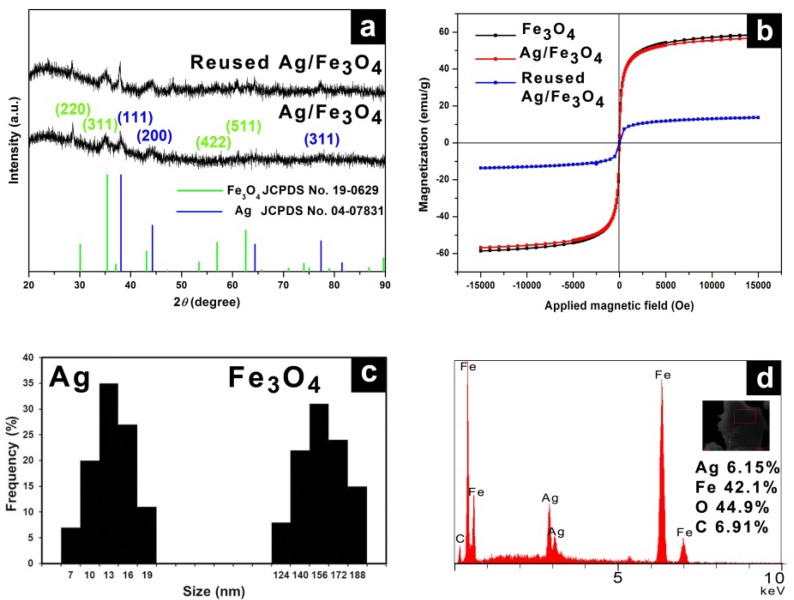
(**a**) XRD pattern of Ag/Fe_3_O_4_ and reused Ag/Fe_3_O_4_; (**b**) SQUID data; (**c**) Size distributions of Ag NPs and Fe_3_O_4_ microspheres; and (**d**) EDS spectrum of Ag/Fe_3_O_4_.

The Fe_3_O_4_ microspheres were synthesized using the solvothermal method [31]. The partial reduction of FeCl_3_ was done at 200 °C using ethylene glycol as a solvent, sodium acetate as a reducing agent, and Na_3_Cit as an electrostatic stabilizer. No pretreatment procedures such as attaching functional groups and coating polymers or carbon onto Fe_3_O_4_, were required prior to the immobilization of Ag NPs onto the Fe_3_O_4_ microspheres. In fact, Ag NPs were easily immobilized on the surface of Fe_3_O_4_ *in situ* because of the interaction between the Fe atoms and carboxyl groups of Na_3_Cit with Ag. Figure 1 illustrates the morphology of the Ag/Fe_3_O_4_ catalyst. Figure 1a shows a scanning electron microscopy (SEM) image of spherical Fe_3_O_4_ microspheres. The immobilized Ag NPs on Fe_3_O_4_ microspheres were observed in the transmission electron microscopy (TEM) images (Figure 1b–d). The X-ray diffractometer (XRD) pattern of the Ag/Fe_3_O_4_ and reused Ag/Fe_3_O_4_ microspheres corresponded to a cubic spinel structure of Fe_3_O_4_ (JCPDS No. 19-0629) and face-centered cubic of Ag (JCPDS No. 04-07831) (Figure 2a).

In particular, the crystalline pattern was assigned to the (220), (311), (422) and (511) reflections of Fe_3_O_4_ and (111), (200) and (311) reflections of Ag. The superconducting quantum interference device (SQUID) results in Figure 2b shows the magnetic curves as a function of the applied field at 300 K. The saturation magnetization value of Ag/Fe_3_O_4_ was 58.7 emu∙g^−1^, which was similar to that of the Fe_3_O_4_ microspheres (56.9 emu∙g^−1^). The small decrease of the magnetization value of Ag/Fe_3_O_4_ microspheres compared to that of Fe_3_O_4_ microspheres can be attributed to the slight increase of mass due to the immobilized Ag nanoparticles on the surface of Fe_3_O_4_ microspheres [32]. After hydration, the saturated magnetization value was decreased to 13.7 emu∙g^−1^. Moreover, both the remanence (Mr) and coercivity (Hc) of Fe_3_O_4_ microspheres were close to zero, indicating superparamagnetism. The average diameters of Fe_3_O_4_ microspheres and Ag NPs were 150 nm and 12 nm, respectively, as determined from the TEM images (Figure 2c). The elemental compositions of Ag/Fe_3_O_4_ catalysts were obtained using energy-dispersive X-ray spectroscopy (EDS), showing Ag content (atomic: 6.15% and weight: 17 wt%) (Figure 2d).

### 2.2. Reaction Tests

#### 2.2.1. Reduction of Nitro Compounds

**Figure 3:**
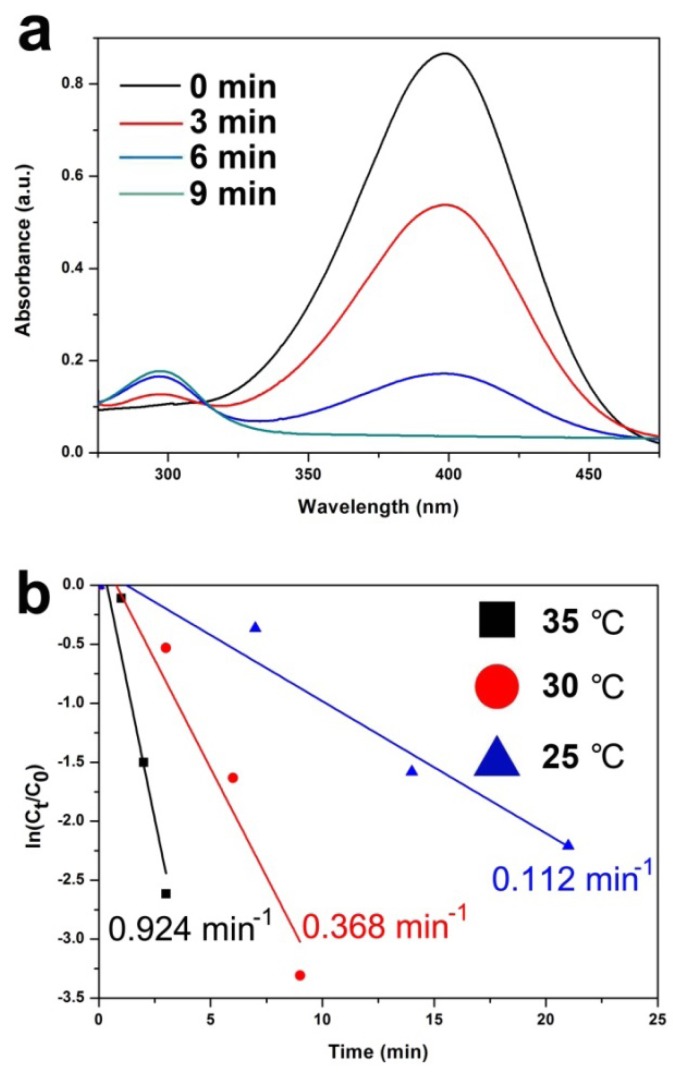
(**a**) Time-dependent UV/vis absorption spectra for the reduction of 4-nitrophenol over hybrid Ag/Fe_3_O_4_ catalyst in aqueous media at 303 K (NaBH_4_: 50 equiv.); and (**b**) Plot of ln(C_t_/C_0_) *versus* time for the reduction of 4-nitrophenol over hybrid Ag/Fe_3_O_4_ catalysts under different temperatures at 2.0 mol% of catalyst and 50 equiv. of NaBH_4_.

As shown in Figure 3a, the UV/Vis spectrum of the reaction mixture was monitored with time during the catalytic reduction of 4-nitrophenol. Specifically, the absorption of 4-nitrophenol at 400 nm decreases rapidly with a concomitant increase in the peak at 300 nm, which is attributed to the reduction product, 4-aminophenol. The control experiments, where only Fe_3_O_4_ microspheres were used as the catalyst, showed no reaction (entry 1, Table 1). In the absence of NaBH_4_, Ag/Fe_3_O_4_ catalyst showed no catalytic activity (entry 2, Table 1). As expected, increasing the number of equivalents of NaBH_4_ increased the catalytic activity (entries 3–5, Table 1). As shown in Figure 3b, the reaction rate constant k is compared under different temperatures at 2.0 mol% of Ag/Fe_3_O_4_ and 50 equiv. of NaBH_4_. The highest catalytic efficiency (0.924 min^−1^) was obtained at 35 °C.

**Table 1:** Optimization of reaction conditions.

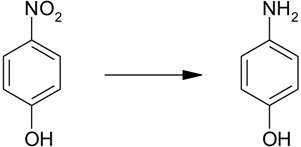					
Entry	Catalyst (mol%)	Temp. (°C)	NaBH_4_ (equiv.)	Time	TOF (h^−1^)
1	Fe_3_O_4_ (2.0)	25	300	4 h	No reaction
2	Ag/Fe_3_O_4_ (2.0)	25	0	4 h	No reaction
3	Ag/Fe_3_O_4_ (2.0)	25	50	20 min	150
4	Ag/Fe_3_O_4_ (2.0)	25	200	3 min	1000
5	Ag/Fe_3_O_4_ (2.0)	25	300	4 min 10 s	1059
6	Ag/Fe_3_O_4_ (2.0)	25	300	2 min 5 s	1440

*Reaction conditions*: 10 mL of 7.50 * 10^−4^ M 4-nitrophenol, 0.026 mg of Ag/Fe_3_O_4_ [Ag base (17 wt%): 2.0 mol%], 1.0 mL of 2.22 M NaBH_4_ (300 equiv. to the substrate).

We then sought to optimize reaction temperature during the reduction (entries 5 and 6, Table 1). The reduction was finished in 2 min 5 s where Ag/Fe_3_O_4_ was used as the catalyst (2.0 mol% of catalyst, 300 equiv. of NaBH_4_ per equiv. substrate). Ag/Fe_3_O_4_ catalyst exhibited superior catalytic activity to previous reported Ag/halloysite nanocomposites and Ag nanoshell-coated cationic polystyrene beads in the comparison with turnover frequency (TOF) value [33,34].

We also confirmed the catalytic activity of hybrid Ag/Fe_3_O_4_ for the reduction of other nitroarene analogues (Table 2). As shown in Table 2, we found that our Ag/Fe_3_O_4_ catalyst promoted high reactivities and excellent yields for a series of model nitrophenols and aniline compounds, regardless of the types and positions of the substituents. 

**Table 2:** Reduction of various nitroarenes using hybrid Ag/Fe_3_O_4_ catalyst.

Entry	Substrate	Product	Time	TOF (h^−1^)
1	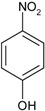	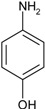	2 min 5 s	1440
2	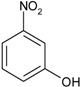	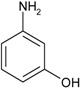	1 min 40 s	1800
3	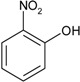	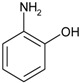	6 min 50 s	439
4	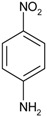	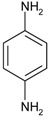	6 min	500
5	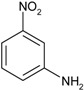	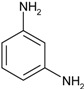	5 min 40 s	529
6	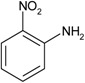	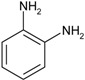	18 min 20 s	164

*Reaction conditions*: 10 mL of 7.50 * 10^−4^ M 4-nitrophenol, 0.026 mg of Ag/Fe_3_O_4_ [Ag base (17 wt%): 2.0 mol%], 1.0 mL of 2.22 M NaBH_4_ (300 equiv. to the substrate) at 308 K.

Interestingly, when the reductions of 4-, 3-, and 2-nitrophenols were catalyzed by Ag/Fe_3_O_4_, the 4- and 3-nitrophenol reductions showed better activities than that of 2-nitrophenol because of a steric effect (entries 1–3, Table 2). Remarkably, the TOF in Entry 2 is 1,800 h^−1^, which is calculated using the moles of nitroarene consumed per mole of the hybrid Ag/Fe_3_O_4_ catalyst for a reaction time of 1 h under the present reaction condition. Interestingly, m-nitro compounds exhibited better conversion efficiencies than other nitro compounds because of a resonance effect (entries 4–6, Table 2).

#### 2.2.2. Hydration of Nitriles to Amides

The reaction of benzonitrile in water was chosen as the test model. A possible mechanism of nitrile hydration by Ag catalyst is shown by Satsuma’s group [28]. Almost no reaction occurred in the absence of a catalyst (entry 1, Table 3). Generally, time, temperature, and quantity of catalyst are important considerations for increasing rate of the conversion. A 2% conversion was obtained at 100 °C within 2 h of the reaction in the presence of 1.0 mol% catalyst (entry 2, Table 3). When the temperature was increased to 150 °C, 25% conversion was achieved within the same reaction time, *i.e.*, 2 h (entry 3, Table 3). As mentioned in entry 4, the conversion increased slightly within 6 h of the reaction. Finally, the optimum reaction conditions were found to be as follows: nitrile (0.1 mL, 1.0 mmol) with Ag/Fe_3_O_4_ (19.0 mg, 3.0 mol%) in H_2_O (3.0 mL) in a stainless steel reactor (entry 5, Table 3). Under these conditions, Ag/Fe_3_O_4_ catalyst showed better catalytic activity than a previously reported SiO_2_-supported Ag nanocatalyst [28]. 27% conversion was obtained with a reaction time of 6 h at 120 °C (entry 6, Table 3).

**Table 3:** Optimization of reaction conditions.

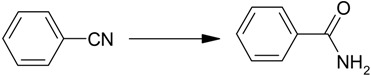				
Entry	Catalyst (mol%)	Temp. (°C)	Time (h)	Conversion ^a^ (%)
1	Blank	150	6	8
2	1	100	2	2
3	1	150	2	25
4	1	150	6	36
5	3	150	6	>99
6	3	120	6	27
7	2	150	6	85
8	3	150	1	31
9	Recovered from #5	150	6	>99
10	Recovered from #9	150	6	>99
11	Recovered from #10	150	6	>99

*^a^* Determined by using gas chromatography-mass spectrometery (GC-MS) spectroscopy. *Reaction conditions*: nitriles (1.0 mmol), Ag/Fe_3_O_4_ catalyst (3.0 mol%), H_2_O (3.0 mL).

**Scheme 2:**
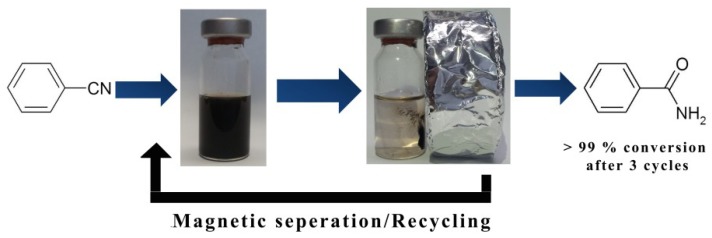
Magnetic separation and recycling of the Ag/Fe_3_O_4_ catalyst.

**Figure 4:**
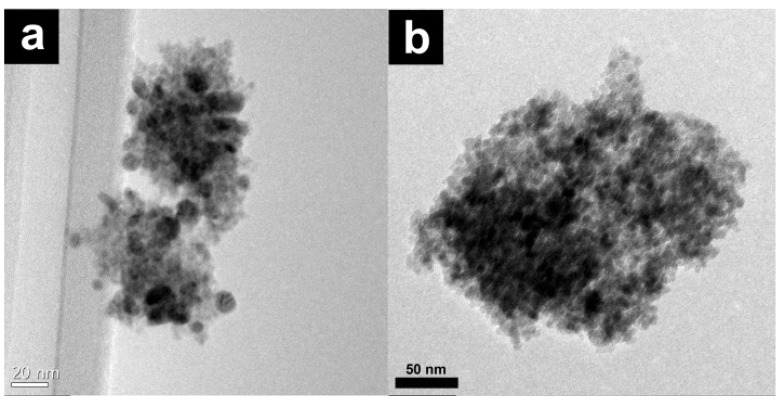
TEM images of Ag/Fe_3_O_4_, (**a**) before and (**b**) after the third cycles.

Interestingly, the conversion decreased only slightly when 2.0 mol% Ag/Fe_3_O_4_ was used (entry 7, Table 3). As expected, lowering the reaction time effectively decreased the conversion (entry 8, Table 3). Among the three factors of reaction time, temperature, and quantity of catalyst, we found that the reaction temperature was the most important because the conversion decreased drastically at 120 °C (entries 5 and 6, Table 3). Remarkably, the Ag/Fe_3_O_4_ catalysts were easily separated using an external magnet (Scheme 2) after the completion of the reaction, and reused three times under the same reaction conditions without any loss of catalytic activity (entries 9–11, Table 3). As shown in Figure 4, the structure of the Ag/Fe_3_O_4_ microspheres remained unchanged after the reaction ended, thereby demonstrating catalyst recyclability.

The optimized reaction conditions determined for the Ag/Fe_3_O_4_ catalyst system were applied to various other substituents (Table 4).

**Table 4:** Hydration of various nitriles catalyzed by Ag/Fe_3_O_4_ catalyst.

Entry	Substrate	Product	Yield ^a^ (%)
1	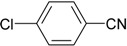	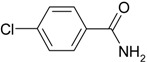	>99
2	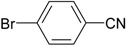	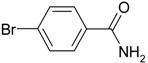	96
3	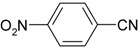	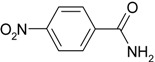	60
4	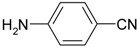	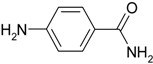	40
5	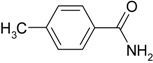	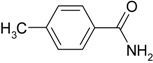	98
6	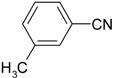	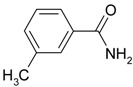	86
7	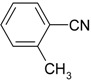	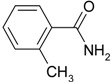	13
8	H_3_C—CH	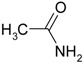	>99
9	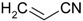	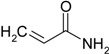	87

*^a^* Determined by using GC-MS Spectroscopy. *Reaction conditions*: nitriles (1.0 mmol), Ag/Fe_3_O_4_ catalyst (3.0 mol%), H_2_O (3.0 mL), 150 °C, 6 h.

We confirmed that this reaction could be extended to a wide variety of nitriles. The reaction rates were not influenced substantially by the electronic effects of the substituents on the aromatic rings of nitriles. Both, 4-chlorobenzonitrile and 4-bromobenzonitrile showed high conversions of more than 95% (entries 1 and 2, Table 4). The nitro and amino groups on the nitrile were less active than the halide substituents (entries 3 and 4, Table 4). When the hydration of *o*-, *m*-, and *p*-tolunitriles was catalyzed by Ag/Fe_3_O_4_, steric effects of *ortho*-substituted nitriles on the reaction rates were observed (entries 5–7, Table 4). The hydration of aliphatic nitriles such as acetonitrile and acrylonitrile was also accomplished with high conversions (entries 8 and 9, Table 4)

## 3. Experimental

### 3.1. General Remarks

The morphology of each sample was characterized by TEM (FEI, Tecnai F30 Super-Twin) located at the National Nanofab Center (Daejeon, South Korea) by placing a few drops of the corresponding colloidal solution on carbon-coated copper grids (200 mesh, F/C coated, Ted Pella Inc., Redding, CA, USA). The SEM images were taken using a SEM (VEGA3, TESCAN, Busan, South Korea). Magnetization data were taken using a SQUID (MPMS-7, Quantum Design, Busan, South Korea). The elemental compositions of the hybrid catalysts were obtained using EDS (550i, IXRF Systems, Inc., Busan, South Korea), while the XRD patterns were recorded by a Rigaku RINT 2200 HK diffractometer (Rigaku Corporation, Tokyo, Japan). Mass spectra were obtained on a Shimadzu GC/MS, QP-2010 SE (EI) (Shimadzu Co., Kyoto, Japan). Reagents were purchased from Aldrich Chemical Co., TCI and Strem Chemical Co. and used as received without further purification. The concentration of 4-nitrophenol was determined at a wavelength of 400 nm using a SINCO S-3150 spectrophotometer (SINCO, Daejeon, Korea).

### 3.2. Synthesis of Fe_3_O_4_ Microspheres

Magnetite particles were synthesized using a solvothermal method [31]. The details were as follows: FeCl_3_•6H_2_O (0.36 g, 1.3 mmol) and trisodium citrate (Na_3_Cit, 0.072 g, 0.24 mmol) were dissolved in ethylene glycol/ethanol (36 mL/4 mL) solution; then, sodium acetate (0.48 g, 5.9 mmol) was added under vigorous stirring for 10 min. The resulting mixture was then transferred to a Teflon-lined stainless-steel autoclave (with a capacity of 50 mL) for heating at 200 °C for 10 h. Then, the autoclave was carefully taken out and allowed to cool to room temperature. The as-made black products were thoroughly washed with ethanol three times, and they were then vacuum-dried. 

### 3.3. Immobilization of Ag NPs onto Fe_3_O_4_ Microspheres

Ag NPs were immobilized onto Fe_3_O_4_ microspheres according to modified procedure of Yang *et al.* [31]. Fe_3_O_4_ microspheres were incubated in 0.2 M NaOH aqueous solution to ionize the carboxyl groups. The residual NaOH was removed by washing with deionized water through centrifugation. Then, the ionized Fe_3_O_4_ microspheres were dispersed in the solution (30 mL, 0.1 M) of AgNO_3_ during 1 h under sonication. After this, the microspheres were harvested with the aid of the magnet and washed with deionized water three times. Then, the microspheres were redispersed in 20 mL of deionized water, and 50 mM NaBH_4_ aqueous solution (1 mL) was added dropwise under ice water bath cooling during 5 min with vigorous stirring. The final product was purified through washing with water three times and dried under vacuum.

### 3.4. A Typical Procedure for Reduction of Nitroarenes

As a representative example, 7.50 × 10^−4^ M 4-nitrophenol solution and 0.026 mg of Ag/Fe_3_O_4_ [Ag base (17 wt%): 2.0 mol%] were mixed and sonicated for 30 s at room temperature. Then, 1.0 mL of 2.22 M NaBH_4_ (300 equiv. to per equiv. of substrate) solution was added to the mixture. The reaction progress was monitored by UV/vis spectrometer.

### 3.5. A Typical Procedure for the Hydration of Nitriles

Ag/Fe_3_O_4_ catalyst (3.0 mol%), water (3.0 mL), and the corresponding nitrile (1.0 mmol) were introduced into a stainless steel reactor. After the reaction, the catalysts were separated from the solution by external magnet. The reaction products were analyzed by mass spectra on GC-MS. (Figures S1–S10).

#### 3.5.1. GC-MS Data

Benzamide (Table 3, entry 5). To a stainless steel reactor equipped with magnetic stirrer were added benzonitrile 0.1 mL (1.0 mmol), Ag/Fe_3_O_4_ 19.0 mg (0.03 mmol) and H_2_O (3 mL). The mixture was heated at 150 °C for 6 h. After cooling to room temperature, the solution was extracted with ethylacetae (20 mL). MS (EI) *m/z*: 28(100), 32(34), 51(30), 77(74), 105(75), 121(60).

4-Chlorobenzamide (Table 4, entry 1). 4-Chlorobenzonitrile (138 mg, 1.0 mmol) was hydrolysed as above. MS (EI) *m/z*: 75(33), 111(52), 139(100), 155(51).

4-Bromobenzamide (Table 4, entry 2). 4-Bromobenzonitrile (182 mg, 1.0 mmol) was hydrolysed as above. MS (EI) *m/z*: 28(27), 50(48), 139(100), 155(51), 183(100), 199(51).

4-nitrobenzamide (Table 4, entry 3). 4-nitrobenzonitrile (148 mg, 1.0 mmol) was hydrolysed as above. MS (EI) *m/z*: 28(100), 118(28), 150(78), 166(55).

4-aminobenzamide (Table 4, entry 4). 4-aminobenzonitrile (118 mg, 1.0 mmol) was hydrolysed as above. MS (EI) *m/z*: 28(100), 32(33), 65(29), 92(31), 120(75), 136(55).

4-Methylbenzamide (Table 4, entry 5). 4-Methylbenzonitrile (117 mg, 1.0 mmol) was hydrolysed as above. MS (EI) *m/z*: 28(85), 32(28), 65(25), 91(72), 119(100), 135(62).

3-methylbenzamide (Table 4, entry 6). 3-methylbenzonitrile (117 mg, 1.0 mmol) was hydrolysed as above. MS (EI) *m/z*: 65(26), 91(83), 119(100), 135(71).

2-methylbenzamide (Table 4, entry 7). 2-methylbenzonitrile (117 mg, 1.0 mmol) was hydrolysed as above. MS (EI) *m/z*: 65(33), 91(100), 119(87), 135(84 ).

Acetamide (Table 4, entry 8). acetonitrile (0.052 mL, 1.0 mmol) was hydrolysed as above. MS (EI) *m/z*: 28(100), 32(32), 44(77), 59(89).

Acrylamide (Table 4, entry 9). acrylonitrile (0.066 mL, 1.0 mmol) was hydrolysed as above. MS (EI) *m/z*: 27(75), 28(73), 32(17), 43(31), 74(83), 55(64), 71(100).

## 4. Conclusions

Ag NPs were immobilized onto Fe_3_O_4_ microspheres *in situ* by substituting sodium cations with Ag ions. The Ag NPs were uniformly immobilized in the Fe_3_O_4_ support while preserving their particle size and crystallinity, as well as the structural integrity of the Fe_3_O_4_ support. The Ag/Fe_3_O_4_ catalyzed the reduction of nitro compounds and hydration of nitriles to amides in water with high conversion. Furthermore, the Ag/Fe_3_O_4_ catalyst was readily separated using an external magnet and could be reused at least three times with benzonitrile under the optimized reaction conditions without any loss of catalytic activity. The magnetic separability eliminated the requirement of catalyst filtration after the completion of the reaction.

## Supplementary Materials

Supplementary materials can be accessed at: http://www.mdpi.com/1420-3049/19/1/699/s1.
