# Supplementary material for: Magnetically Separable and Recyclable Fe3O4-Supported Ag Nanocatalysts for Reduction of Nitro Compounds and Selective Hydration of Nitriles to Amides in Water
Source: Molecules. 2014 Jan 7;19(1):699–712. doi: 10.3390/molecules19010699 (PMC6271850; doi:10.3390/molecules19010699)
Supplement: Supplementary file 1 [file molecules-19-00699-s001.pdf]

Supporting Information

GC-MS Data

Figure S1. Benzamide (Table 3, entry 5). (**a**) Retention time-intensity graph; and (**b**) *m/z* value-intensity graph.

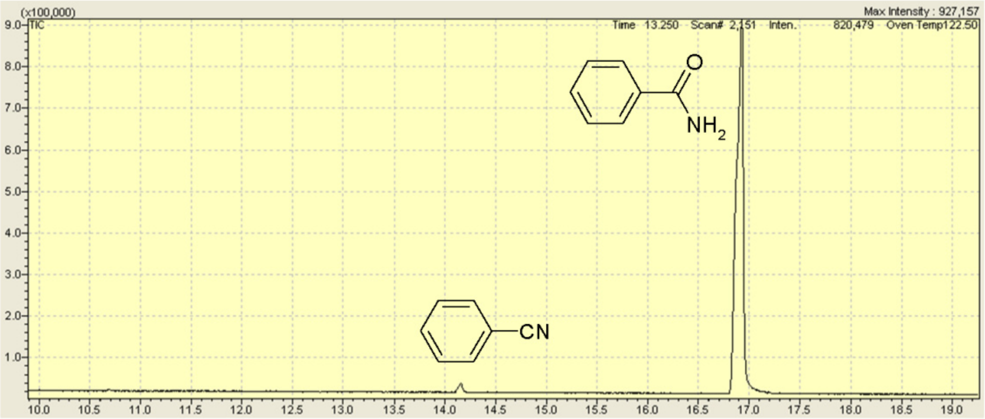

(**a**)

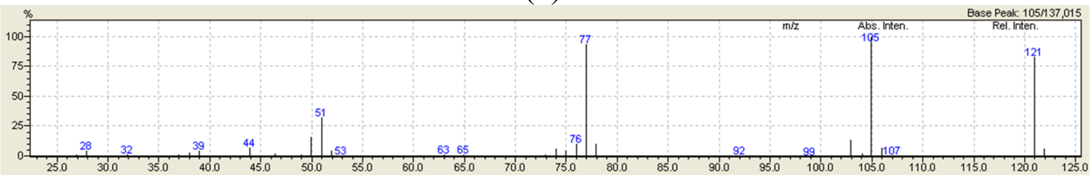

(**b**)

Figure S2. 4-Chlorobenzamide (Table 4, entry 1). (**a**) Retention time-intensity graph; and (**b**) *m/z* value-intensity graph.

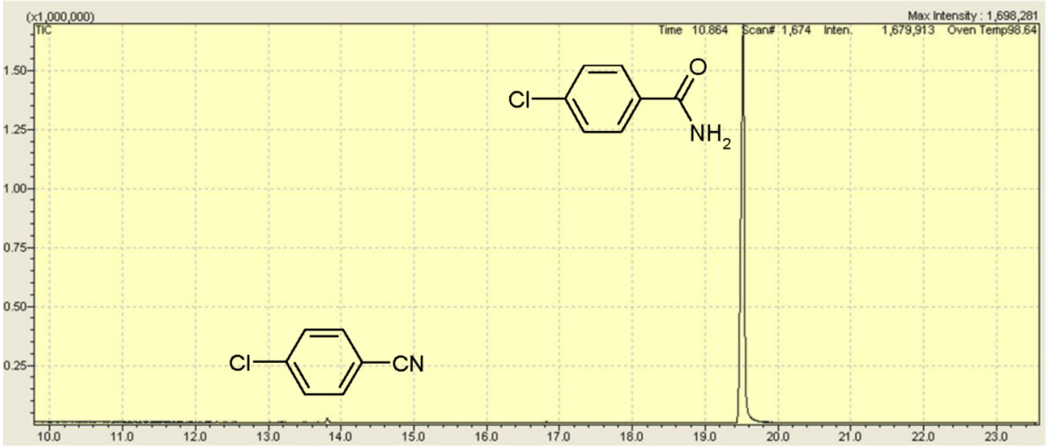

(**a**)

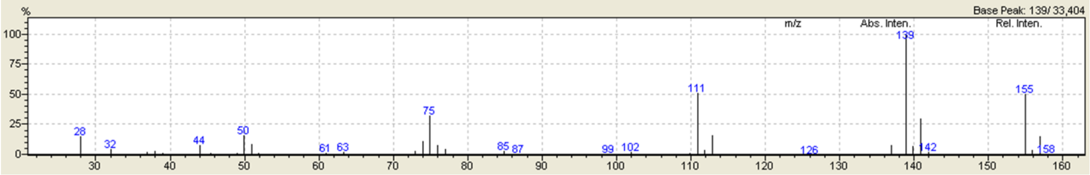

(**b**)

Figure S3. 4-Bromobenzamide (Table 4, entry 2). (**a**) Retention time-intensity graph; and (**b**) *m/z* value-intensity graph.

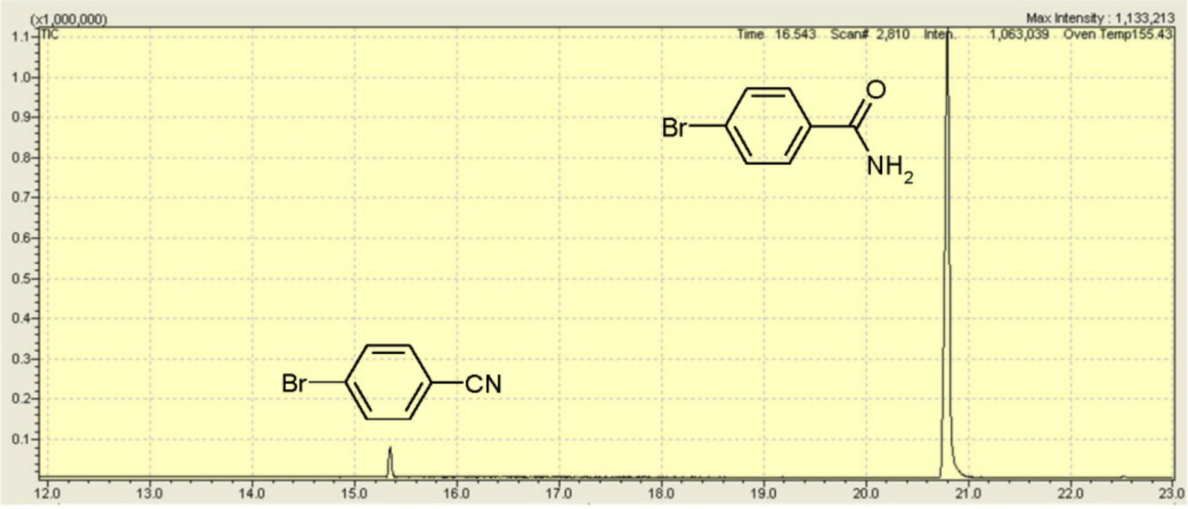

(**a**)

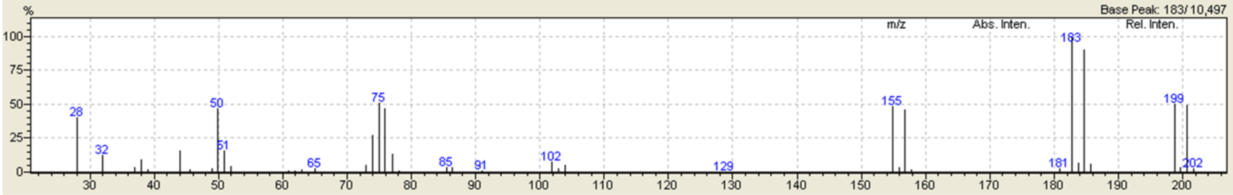

(**b**)

Figure S4. 4-nitrobenzamide (Table 4, entry 3). (**a**) Retention time-intensity graph; and (**b**) *m/z* value-intensity graph.

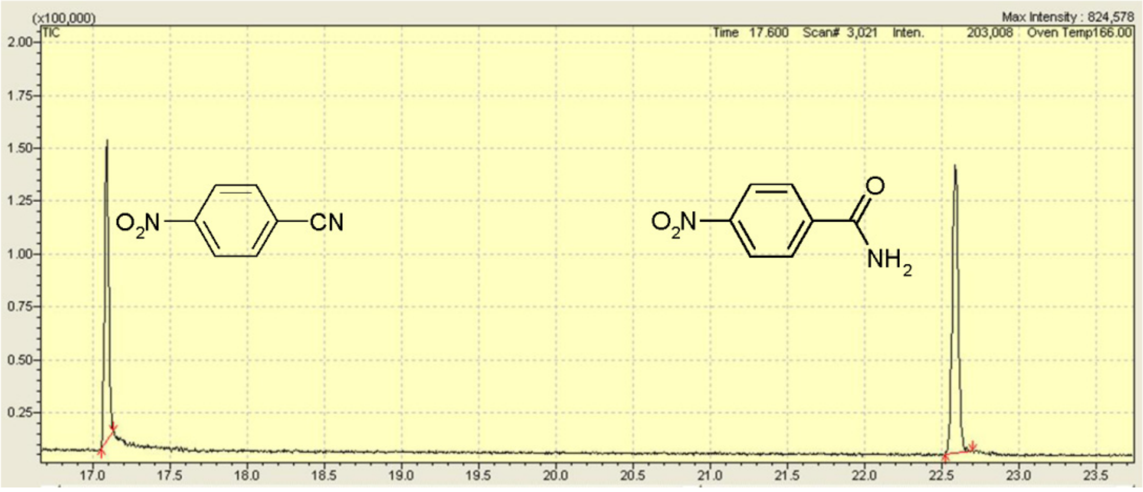

(**a**)

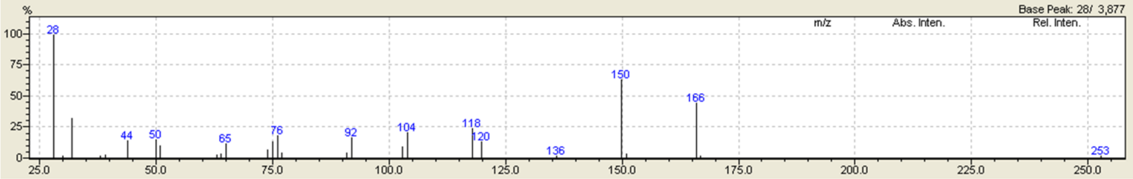

(**b**)

Figure S5. 4-aminobenzamide (Table 4, entry 4). (**a**) Retention time-intensity graph; and (**b**) *m/z* value-intensity graph.

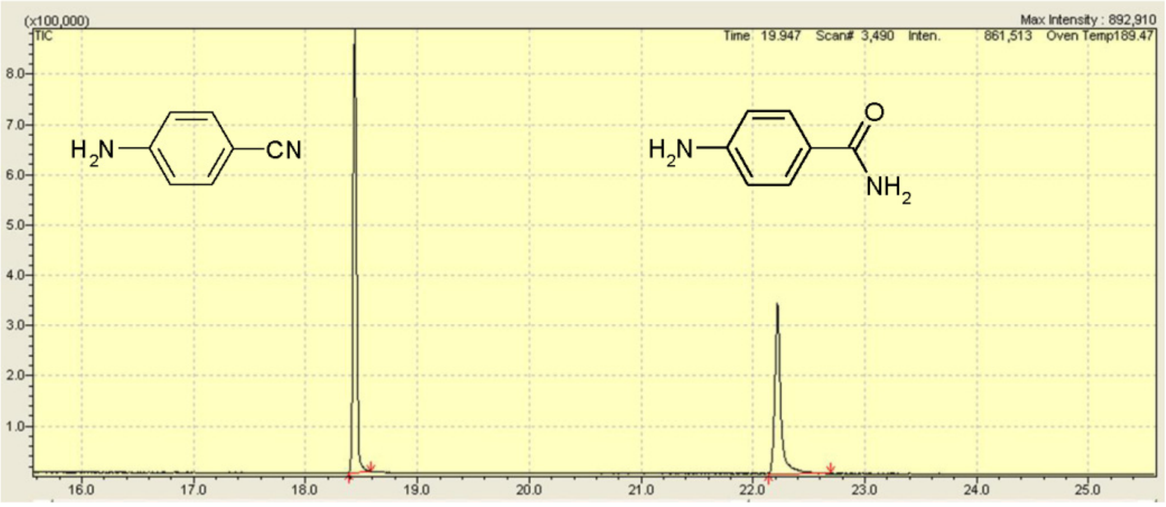

(**a**)

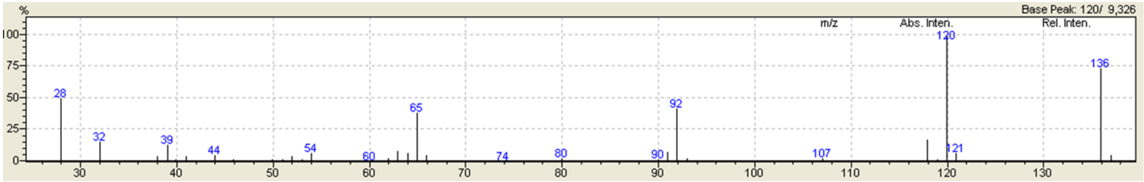

(**b**)

Figure S6. 4-Methylbenzamide (Table 4, entry 5). (**a**) Retention time-intensity graph; and (**b**) *m/z* value-intensity graph.

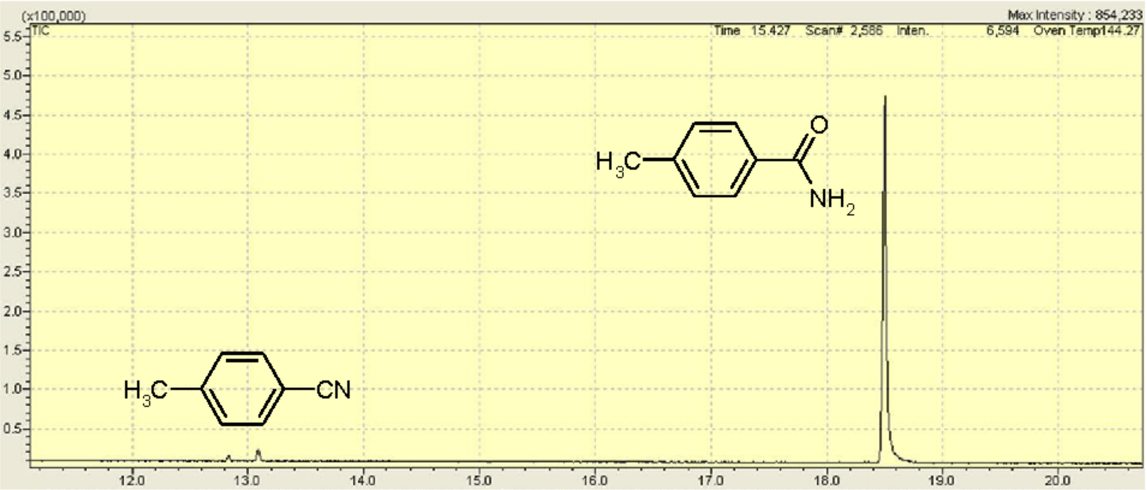

(**a**)

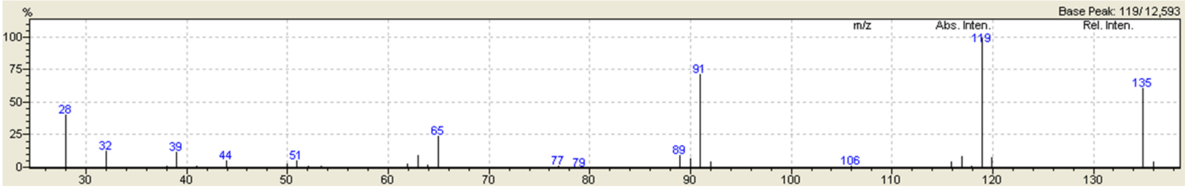

(**b**)

Figure S7. 3-methylbenzamide (Table 4, entry 6). (**a**) Retention time-intensity graph; and (**b**) *m/z* value-intensity graph.

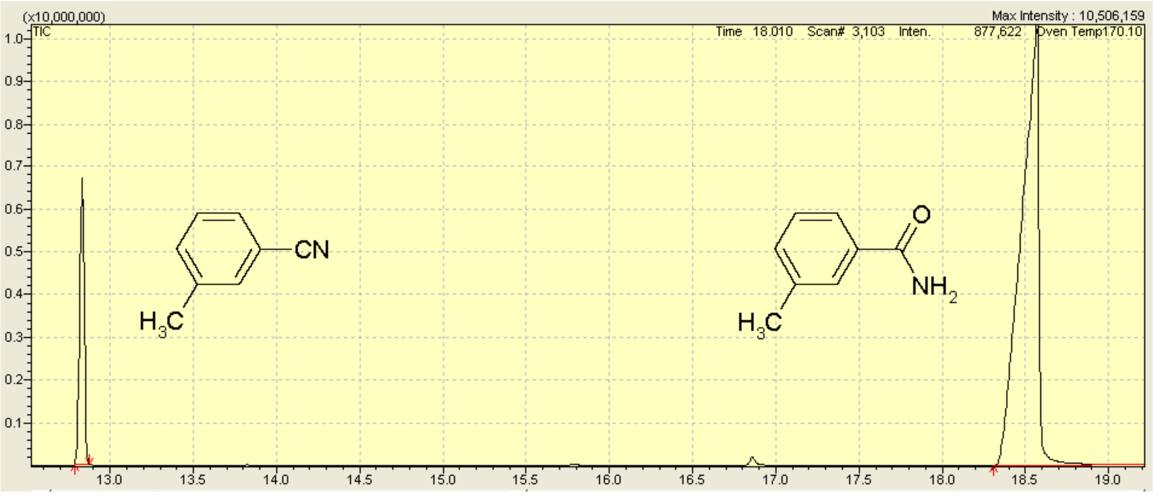

(**a**)

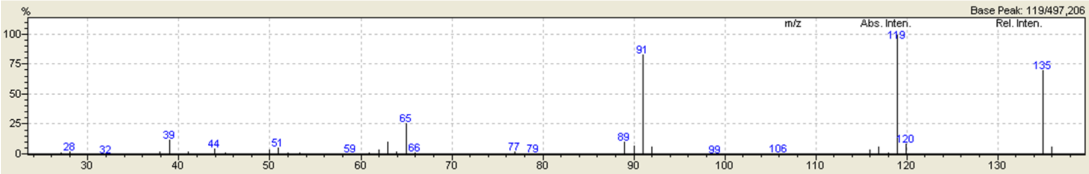

(**b**)

Figure S8. 2-methylbenzamide (Table 4, entry 7). (**a**) Retention time-intensity graph; and (**b**) *m/z* value-intensity graph.

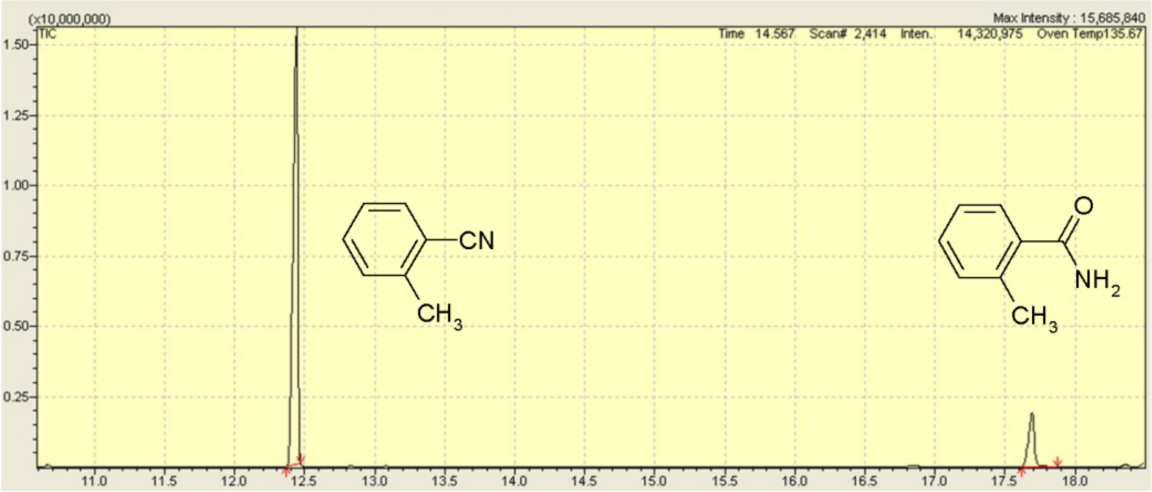

(**a**)

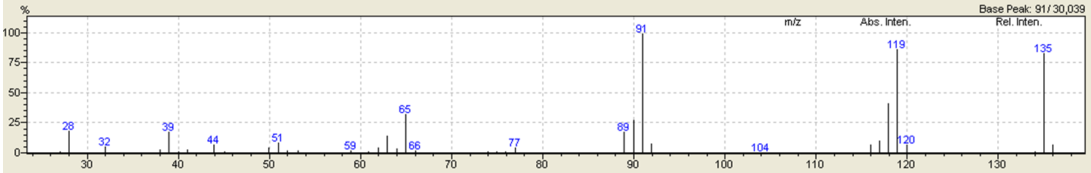

(**b**)

Figure S9. Acetamide (Table 4, entry 8). (**a**) Retention time-intensity graph; and (**b**) *m/z* value-intensity graph.

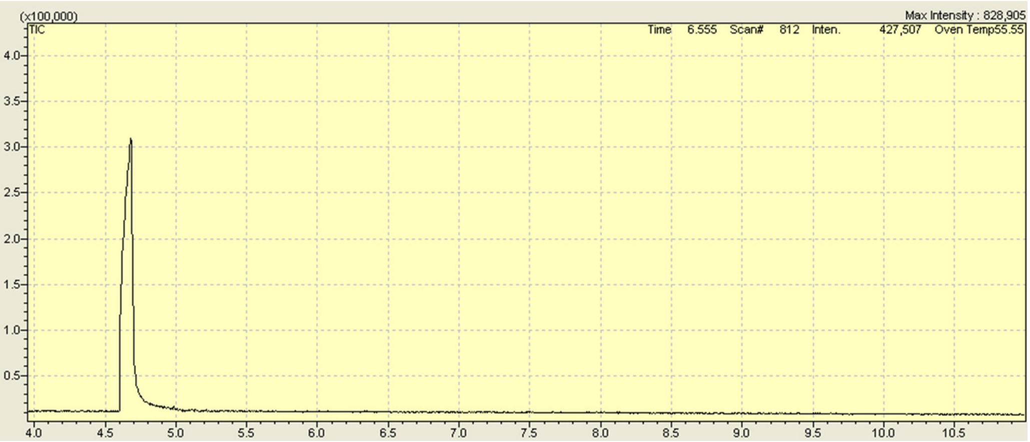

(**a**)

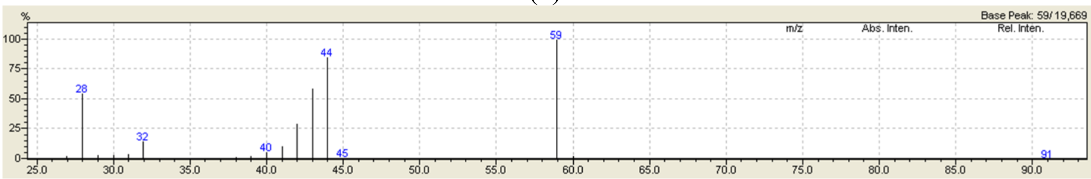

(**b**)

Figure S10. Acrylamide (Table 4, entry 9). (**a**) Retention time-intensity graph; and (**b**) *m/z* value-intensity graph.

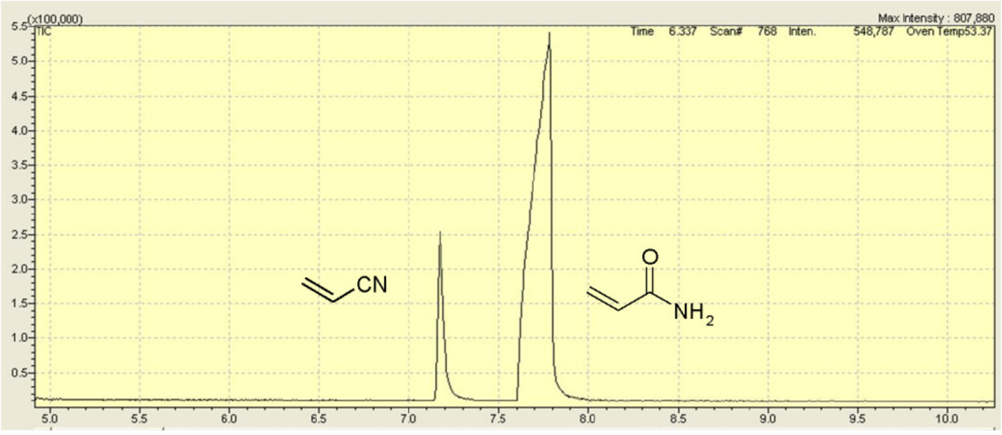

(**a**)

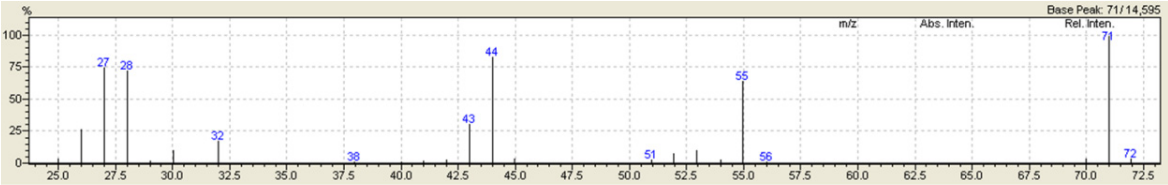

(**b**)
